# Radiomics Based on T2-Weighted Imaging and Apparent Diffusion Coefficient Images for Preoperative Evaluation of Lymph Node Metastasis in Rectal Cancer Patients

**DOI:** 10.3389/fonc.2021.671354

**Published:** 2021-05-10

**Authors:** Chunli Li, Jiandong Yin

**Affiliations:** ^1^ Department of Radiology, Shengjing Hospital of China Medical University, Shenyang, China; ^2^ Department of Biomedical Engineering, School of Fundamental Sciences, China Medical University, Shenyang, China

**Keywords:** rectal cancer, magnetic resonance imaging, radiomics, lymph node metastasis, machine learning

## Abstract

**Purpose:**

To develop and validate a radiomics nomogram based on T2-weighted imaging (T2WI) and apparent diffusion coefficient (ADC) features for the preoperative prediction of lymph node (LN) metastasis in rectal cancer patients.

**Materials and Methods:**

One hundred and sixty-two patients with rectal cancer confirmed by pathology were retrospectively analyzed, who underwent T2WI and DWI sequences. The data sets were divided into training (n = 97) and validation (n = 65) cohorts. For each case, a total of 2,752 radiomic features were extracted from T2WI, and ADC images derived from diffusion-weighted imaging. A two-sample *t*-test was used for prefiltering. The least absolute shrinkage selection operator method was used for feature selection. Three radiomics scores (rad-scores) (rad-score 1 for T2WI, rad-score 2 for ADC, and rad-score 3 for the combination of both) were calculated using the support vector machine classifier. Multivariable logistic regression analysis was then used to construct a radiomics nomogram combining rad-score 3 and independent risk factors. The performances of three rad-scores and the nomogram were evaluated using the area under the receiver operating characteristic curve (AUC). Decision curve analysis (DCA) was used to assess the clinical usefulness of the radiomics nomogram.

**Results:**

The AUCs of the rad-score 1 and rad-score 2 were 0.805, 0.749 and 0.828, 0.770 in the training and validation cohorts, respectively. The rad-score 3 achieved an AUC of 0.879 in the training cohort and an AUC of 0.822 in the validation cohort. The radiomics nomogram, incorporating the rad-score 3, age, and LN size, showed good discrimination with the AUC of 0.937 for the training cohort and 0.884 for the validation cohort. DCA confirmed that the radiomics nomogram had clinical utility.

**Conclusions:**

The radiomics nomogram, incorporating rad-score based on features from the T2WI and ADC images, and clinical factors, has favorable predictive performance for preoperative prediction of LN metastasis in patients with rectal cancer.

## Introduction

More than 700,000 people worldwide were newly diagnosed with rectal cancer in 2018 (1). Among the metastatic pathways of rectal cancer, lymph node (LN) metastasis is the most important and closely correlated with the poor prognosis due to a high rate of local recurrence (2–4). According to the Union for International Cancer Control (UICC) TNM staging classification (8th edition) and the European Society for Medical Oncology (ESMO) Clinical Practice Guidelines, LN status in rectal cancer is an important clinical marker in deciding TNM staging and choosing treatment options within TNM risk category of primary rectal cancer without distant metastases (5, 6). Thus, preoperative assessment of LN metastasis can provide important information to determine the need for adjuvant therapy and the adequacy of surgical resection (5, 7, 8). High-resolution magnetic resonance imaging (MRI) has been widely used for clinical staging and guiding the treatment of rectal cancer patients (9). However, MRI has limited ability to predict LN status with morphological criteria (10, 11). This limitation is aggravated by the lack of consensus on appropriate criteria to assess LN positivity (12). Therefore, improvements in techniques for preoperatively identifying LN metastasis status are key imperatives.

Radiomics based on advanced pattern recognition tools has been considered useful to extract a large number of quantitative features from medical images (13–16). It can provide more metabolic and biological information than conventional imaging methods (17). Previous studies have shown that radiomic features derived from MRI or computed tomography (CT) data have the potential to predict LN metastasis in such malignancies as breast cancer, cervical cancer, and bladder cancer (18–20). For the rectal cancer, some previous studies demonstrated that the histogram features from T2-weighted imaging (T2WI) and the texture features from apparent diffusion coefficient (ADC) maps can help predict lymph node metastasis (21, 22). However, those studies were conducted with comparable or smaller patient sample sizes, focusing mostly on a single-slice image with lower-order histogram or texture features. A recent study in 2021 reported that radiomics analysis based on the single-slice high-resolution T2WI images presented potential in predicting lymph node metastasis of rectal cancer (23). In addition, a study by Liu et al. showed that a radiomics model derived from volume features of T2WI and ADC images achieved excellent performance for the prediction of pathologic complete response in locally advanced rectal cancer (LARC) (24). To the best of our knowledge, there are few studies of radiomics analyses based on multiparametric sequences to identify preoperative LN status in patients with rectal cancer, especially using the features derived from volume lesion of T2WI and ADC images as well as clinical information (25, 26).

Thus, in the current study, we first sought to construct a radiomics score (rad-score) based on features from volume lesion of T2WI and ADC images to distinguish between LN-positive and -negative rectal cancer patients and analyze the discriminative abilities of each imaging model. Then we sought to develop and validate a radiomics nomogram that would incorporate a rad-score based on the combination of T2WI and ADC features, and clinical risk factors to facilitate noninvasive estimation of LN status.

## Materials and Methods

### Patients

The study was approved by the Ethics Review Board of Shengjing Hospital of China Medical University (2020PS011K), and written informed consent was obtained from each patient. In the present study, 236 patients with pathological confirmation of LN status were preliminarily enrolled between September 2018 and August 2020. Seventy-four patients were excluded for the following reasons: (1) patients underwent any treatment before MRI scanning, such as neoadjuvant chemoradiotherapy, endoscopic biopsy, surgery, and so on; (2) image quality was poor due to apparent motion artifacts on the DWI and T2WI sequences. Finally, 162 eligible patients were selected for subsequent analyses. The patients were randomly divided into the training (n = 97) and validation (n = 65) cohorts.

### Histopathologic Assessment

The histopathological evaluation of regional LN malignancy was regarded as the gold-standard for LN metastasis. Pathological reports of surgically resected specimens were retrospectively collected from our PACS. The LN was defined as positive when the number of regional LN metastasis was greater than or equal to one, while the absence of regional LN metastasis was recognized as negative.

### MRI Data Acquisition

All MRI examinations were performed in the supine position on a 3.0-Tesla (T) scanner (Ingenia 3.0, Philips Medical System, Best, The Netherlands) with an eight-channel phased-array surface coil. There was no bowel preparation or intravenous antispasmodic agents administered. High-resolution rectal MRI protocols included transverse DWI and T2WI, and sagittal fat-suppression T2WI. The acquisition parameters for transverse T2WI included: repetition time (TR)/echo time (TE), 2200/65 ms; flip angle, 90°; matrix size, 288 × 288; field of view (FOV), 250 × 250 mm^2^; slices, 20; slice thickness, 5 mm; spacing between slices, 0.5 mm; and NSA, 2. The parameters for DWI included: TR/TE, 6000/76 ms; flip angle, 90°; matrix size, 288 × 288; FOV, 450 × 450 mm^2^; slices, 48; slice thickness, 5 mm; spacing between slices, 1 mm; and b values, 0 and 1,000 s/mm^2^.

DWI and T2WI images were exported from the Picture Archiving and Communication System at our institution. ADC maps were generated using MATLAB 2018a (Mathworks, Natick, MA, United States) according to loaded DWI images using the following formula: ADC = (lnSI_0_-lnSI)/(b-b_0_), where SI_0_ and SI represent signal intensity at b values of 0 and 1,000 s/mm^2^, respectively.

### Tumor Segmentation and Feature Extraction

**Figure 1:**
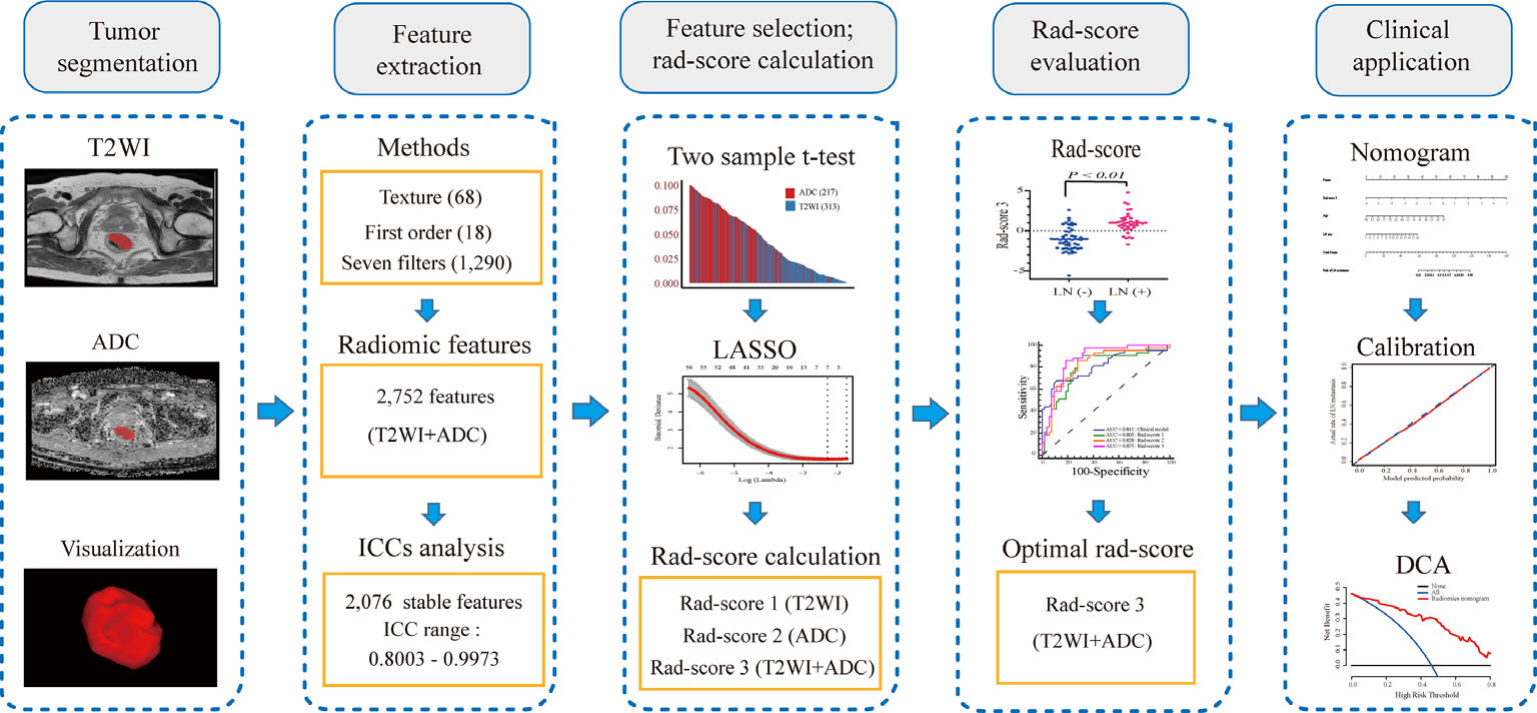
The framework for the radiomics workflow.

Three-dimensional volume of interest (VOI), including the whole tumor and excluding obvious necrosis, hemorrhage, gas, and lumen content areas, was independently segmented on T2WI and DWI data by a radiologist (Reader 1 with 10 years of experience in rectal cancer imaging), who was blinded to the clinical and pathological outcomes. All VOIs were delineated with an open-source software, ITK-SNAP version 3.8.0 from UPenn (http://www.itksnap.org) (27). For T2WI data, the contour of the tumor was manually drawn on each transverse slice. Then, the corresponding VOI was automatically generated by the ITK-SNAP software. For ADC maps, the contour of the tumor was manually delineated along the border of the high signal region on each transverse DWI slice (b-value of 1,000 s/mm^2^) first with reference to T2WI, and then automatically turned into the VOI which was copied to the corresponding ADC maps finally (24). An overview of the radiomics analysis workflow is shown in **Figure 1**.

PyRadiomics, an open-source python package for enabling the standardization of image processes and extracting a large panel of radiomic features from medical images, was used to extract radiomic features from T2WI and ADC data within manually segmented VOIs (28). To avoid data heterogeneity bias of the images, all MRI data were subjected to imaging normalization (the intensity of the image was scaled to 0–100) and resampled to the same resolution (3 mm × 3 mm × 3 mm) before feature extraction (29). In addition, the segmented images were also resampled (3 mm × 3 mm × 3 mm) to maintain VOI accuracy. For each sequence, first-order statistics, texture, and seven built-in filter features (square, square root, logarithm, exponential, gradient, Laplacian of Gaussian [LOG], wavelet) were calculated, which resulted in a total of 1,376 radiomic features, as shown in **Supplementary Table S1**.

We randomly chose 30 cases of MRI images (T2WI and ADC); VOI segmentation was performed by two radiologists (Reader 1, and Reader 2 with 8 years of experience in rectal cancer imaging). Feature extraction was performed on the two sets of VOIs generated by the two radiologists to obtain two groups of the radiomic features. Intraclass correlation coefficients (ICCs) were determined using the two sets of radiomic features to evaluate the reproducibility and stability of each feature. We interpreted a coefficient of 0.81 to 1.00 as an almost perfect agreement, 0.61 to 0.80 as a substantial agreement, 0.41 to 0.60 as a moderate agreement, 0.21 to 0.40 as a fair agreement, and 0 to 0.20 as a poor or no agreement (30). Features with ICC value > 0.8 were collected for subsequent analysis, which were individually subtracted by the mean value of each feature and divided by the respective standard deviation values (Z-score normalization), thus, removing the limitations imposed by the units of each feature (31).

### Feature Selection and Rad-Score Calculation

To reduce the feature dimension and remove irrelevant features, two steps were applied for feature selection. First, some features based on univariate statistical tests (two-sample *t*-test) between LN-positive and -negative groups in the training cohort were selected (24). Second, the least absolute shrinkage and selection operator (LASSO) method (31, 32), which is suitable for the regression of high-dimensional data, was performed within each set of ADC and T2WI data, respectively. The support vector machine (SVM) classifier was used to identify LN metastasis where the kernel parameter was set to the linear kernel, and other parameters were set to default (24). Rad-score 1 and rad-score 2 were calculated for each patient using the SVM model with linear kernel training based on the selected T2WI and ADC features, respectively. For the combination of two sequences, the selected T2WI and ADC features were combined and once more fed into the LASSO method. Accordingly, rad-score 3 was calculated using the SVM model with linear kernel training based on selected fusion features. Feature selection and rad-score calculation were conducted with R software (version 3.6.2, https://www.R-project.org).

### Radiomics Nomogram Development

Univariate logistic regression analysis was first conducted with the following clinical information: age, sex, LN size (maximum LN short diameter), tumor size, tumor location, T stage, and rad-score 3 to identify potential predictors (21). Then multivariate logistic regression analysis was used to select the independent predictors of LN metastasis (21). Based on the multivariable logistic analysis, the clinical model and radiomics nomogram for LN metastasis prediction were constructed with the selected predictors. Calibration curves were used to evaluate the calibration of the radiomics nomogram. The Hosmer–Lemeshow test was conducted to assess the goodness-of-fit of the nomogram. The discrimination performances of the clinical model, three rad-scores, and the radiomics nomogram for predicting LN metastasis were evaluated according to the area under the receiver operator characteristic (ROC) curve (AUC) in both the training and validation cohorts. Decision curve analysis (DCA) was performed to determine the clinical usefulness of the nomogram by quantifying the net benefits at different threshold probabilities in the validation cohort (33). ROC curves were drawn using the professional medical statistics software, MedCalc (version 14.10.20, https://www.medcalc.org/). Calibration and DCA curves were generated using R software.

### Statistical Analysis

Univariate analysis was used to compare the differences in clinical and pathological characteristics between LN-positive and -negative groups using the chi-square test for categorical variables, and two-sample *t*-tests for continuous variables, as appropriate. All statistical tests were two-tailed and were conducted with a statistical significance level of 0.05. Statistical analyses were performed and figure plots were generated with R software and SPSS software (SPSS Inc., Chicago, IL). The DeLong test was used to statistically compare the AUCs between the models.

## Results

### Clinical and Pathological Characteristics

Patient characteristics were summarized in **Table 1**. Age, LN size (maximum LN short diameter), and T stage were significantly different between the LN-positive and -negative groups. There were no significant differences in other clinical characteristics (sex, tumor size, and tumor location) between the LN-positive and -negative groups. No difference in the LN positive rate was observed between the two cohorts (44.3% (43/97) vs. 46.2% (30/65), respectively; *P* = 0.819).

**Table 1:** Clinical and pathological features of patients.

Characteristic	Training cohort, n = 97		*P*	Validation cohort, n = 65		*P*
	LN (+), n = 43	LN (–), n = 54		LN (+), n = 30	LN (-), n = 35	
Age, year	55.6 ± 13.3	65.2 ± 7.4	*<0.01^a^*	56.8 ± 11.9	63.8 ± 10.7	*0.016*
Sex (%)			*0.146*			*0.347*
Male	25 (58.1%)	39 (72.2%)		20 (66.7%)	27 (77.1%)	
Female	18 (41.9%)	15 (27.8%)		10 (33.3%)	8 (22.9%)	
LN size (mm)	6.6 ± 2.8	5.0 ± 2.1	*<0.01^a^*	6.9 ± 2.5	4.9 ± 1.8	*<0.01^a^*
Tumor size (cm)	4.5 ± 1.2	4.7 ±1.2	*0.434*	4.6 ± 1.3	4.9 ± 1.4	*0.385*
Tumor location (cm)	6.1 ± 1.2	6.3 ± 2.5	*0.567*	6.6 ± 2.0	7.0 ± 2.9	*0.454*
T stage (%)			*0.023*			*<0.01^a^*
T1-2	7 (16.3%)	20 (37.0%)		4 (13.3%)	16 (45.7%)	
T3-4	36 (83.7%)	34 (63.0%)		26 (86.7%)	19 (54.3%)	
Rad-score 1	0.60 ± 1.24	−0.82 ± 1.21	*<0.01^a^*	0.23 ± 0.87	−0.64 ± 1.07	*<0.01^a^*
Rad-score 2	0.64 ± 1.36	−1.07 ± 1.21	*<0.01^a^*	0.29 ± 1.34	−0.85 ± 1.22	*<0.01^a^*
Rad-score 3	0.98 ± 1.20	−1.07 ± 1.39	*<0.01^a^*	0.34 ± 0.99	−0.97 ± 1.02	*<0.01^a^*

P was derived from the univariable association analyses between each of the clinicopathological variables and LN status. Chi-Square was used to compare the differences in categorical variables (sex, T stage), while the two-sample t-test was used to compare differences in age, LN size (maximum LN short diameter), tumor size, tumor location, and rad-scores. ^a^P < 0.05 is considered statistically significant.

Of the 2,752 radiomic features extracted from T2WI and ADC images, 2,076 were demonstrated to have high stability, with ICCs from 0.8003 to 0.9973.

### Feature Extraction, Selection, and Rad-Score Calculation

**Figure 2:**
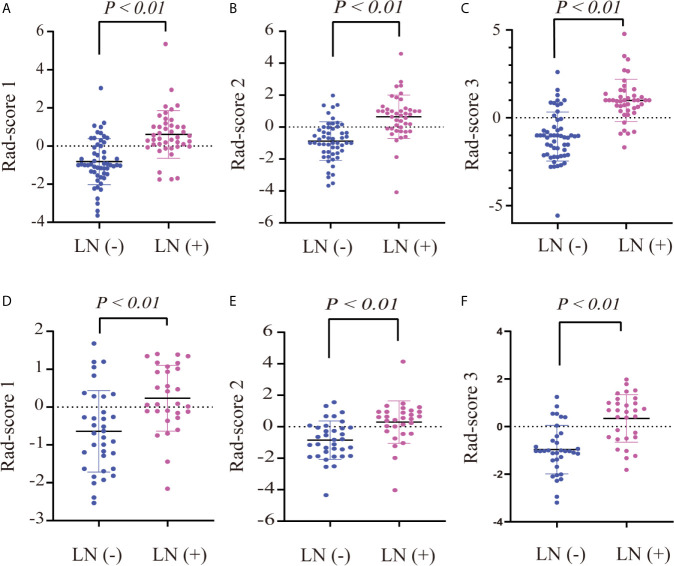
Dot diagram of the three rad-scores in each cohort. Dot diagram of rad-score 1 in the training **(A)** and validation **(D)** cohorts. Dot diagram of rad-score 2 in the training **(B)** and validation **(E)** cohorts. Dot diagram of rad-score 3 in the training **(C)** and validation **(F)** cohorts.

To reduce the number of weak features, we first performed univariate analysis (two-sample *t*-tests) as a feature filter in the training cohort. We included more features than those that showed significant differences between LN-positive and -negative groups as compensation to avoid eliminating highly discriminative features in multivariate analyses, rather than univariate analysis (17). Two-sample *t*-tests (*P* < 0.1) allowed for the selection of 530 features, including 313 T2WI and 217 ADC features. Next, 313 T2WI and 217 ADC features were respectively reduced to seven and 11 potential predictors by applying LASSO logistic regression using 10-fold cross-validation *via* the minimum criteria. Finally, the combination of the seven T2WI and 11 ADC features was reduced to 13 potential predictors by applying LASSO logistic regression using 10-fold cross-validation *via* the minimum criteria. Three rad-scores were calculated. The resultant coefficients of features in each group used in calculating the corresponding rad-score were shown in **Supplementary Table S2**. The distributions of the three rad-scores and LN status in the training and validation cohorts were shown in **Figure 2**.

### Rad-Score Evaluation

There were significant differences in rad-score 1, rad-score 2, and rad-score 3 between LN-positive and -negative patients in the training cohort (*P* < 0.01); the same result was achieved in the validation cohort (*P* < 0.01), as shown in **Table 1**. The dot diagram showed that the three rad-scores for LN-positive patients were generally higher than those for LN-negative patients in the training and validation cohorts (**Figure 2**).

**Figure 3:**
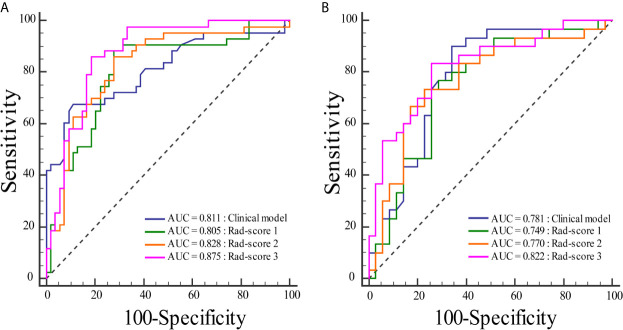
Comparisons of the ROC curves for the clinical model and three rad-scores in each cohort. **(A)** The ROC curves for the clinical model and three rad-scores in the training cohort. **(B)** The ROC curves for the clinical model and three rad-scores in the validation cohort.

To compare the classification performance, the ROC curves were plotted for the clinical model, rad-score 1, rad-score 2, and rad-score 3 in the training and validation cohorts (**Figure 3**). The clinical model achieved an AUC of 0.811 in the training cohort and an AUC of 0.781 in the validation cohort. The AUCs of the rad-score 1 and rad-score 2 were 0.805, 0.749 and 0.828, 0.770 in the training and validation cohorts, respectively. The rad-score 3 yielded the highest AUC scores among four models in both training (0.875) and validation (0.822) cohorts. There was significant difference in AUC between rad-score 1 and rad-score 3 in the training cohort, but not among the other models. No significant difference was found in AUC among four models in the validation cohort. The detailed results were shown in **Table 2**.

**Table 2:** AUC comparison based on DeLong test among four models.

Cohort	Model	Clinical model	Rad-score 1	Rad-score 2	Rad-score 3
Training	Clinical model	/	0.939	0.805	0.302
	Rad-score 1	0.939	/	0.691	0.011
	Rad-score 2	0.805	0.691	/	0.250
	Rad-score 3	0.302	0.011	0.250	/
Validation	Clinical model	/	0.728	0.886	0.601
	Rad-score 1	0.728	/	0.783	0.105
	Rad-score 2	0.886	0.783	/	0.342
	Rad-score 3	0.601	0.105	0.342	/

### Radiomics Nomogram Construction and Evaluation

**Figure 4:**
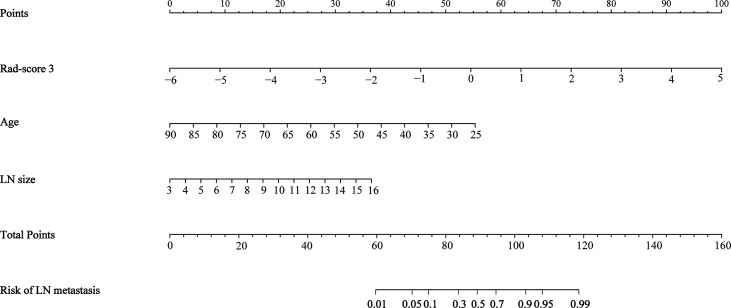
Radiomics nomogram incorporating the rad-score 3, age, and the LN size.

The results of univariate and multivariate logistic regression analysis were provided in **Table 3**. Univariate analysis showed that age, LN size, T stage, and rad-score 3 had significant differences between LN-positive and -negative groups in the training cohort. In multivariate logistic analysis, the rad-score 3, age, and LN size were identified as independent parameters of LN metastasis. A radiomics nomogram, incorporating the age, LN size, and rad-score 3, was developed, as shown in **Figure 4**.

**Table 3:** Univariate and multivariate logistic regression analysis of the clinical parameters and rad-score 3.

Parameters	Univariate analysis		*P*-value	Multivariate analysis		*P*-value	
	OR	95% CI		OR	95% CI		
Age	0.910	0.865–0.957	< 0.01*	0.873	0.803–0.950	< 0.01*	
Sex	1.872	0.801–4.378	*0.148*				
LN size	1.314	1.094–1.580	*0.004*	1.545	1.138–2.099	< 0.01*	
Tumor size	0.870	0.615–1.230	*0.430*				
Tumor location	0.946	0.783–1.142	*0.563*				
T stage	3.025	1.135–8.061	*0.027*	3.915	0.809–18.941	*0.090*	
Rad-score 3	3.582	2.190–5.859	< 0.01*	4.503	2.321–8.735	< 0.01*	

OR, odds ratio; CI, confidence interval. *P-value < 0.05 is considered statistically significant.

**Figure 5:**
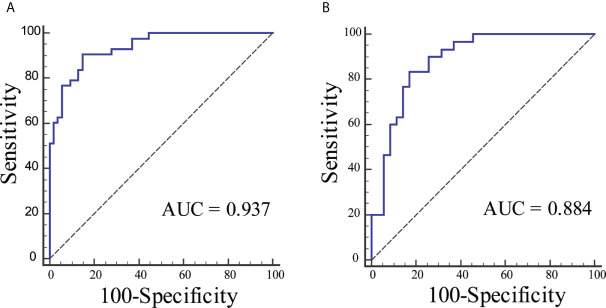
The ROC curves for radiomics nomogram in each cohort. **(A)** The ROC curve for radiomics nomogram in the training cohort. **(B)** The ROC curve for radiomics nomogram in the validation cohort.

**Figure 6:**
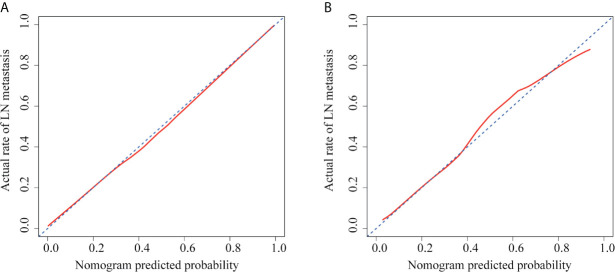
Calibration curves of radiomics nomogram in each cohort. **(A)** The calibration curve of radiomics nomogram in the training cohort. **(B)** The calibration curve of radiomics nomogram in the validation cohort. The x-axis represented the predicted LN metastasis risk. The y-axis represented the actual LN metastasis rate. The diagonal blue line represented a perfect prediction by an ideal model. The red line represented the performance of the radiomics nomogram, of which a closer fit to the diagonal blue line represented a better prediction.

**Figure 7:**
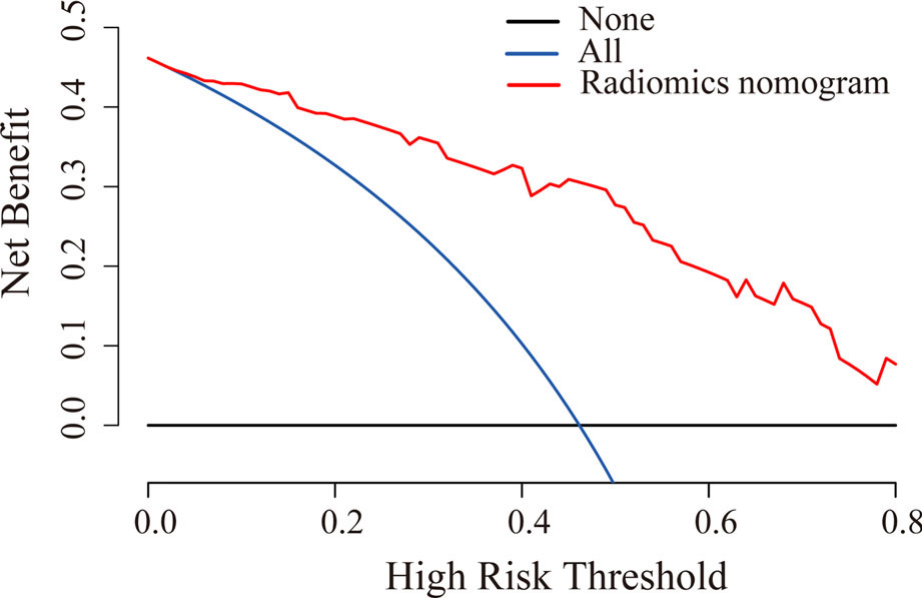
DCA for radiomics nomogram in the validation cohort. The y-axis indicated the net benefit. The red line, blue line, and horizontal black line represented the net benefit of the radiomics nomogram, treat-all strategy, and treat-none strategy, respectively.

The ROC curves were plotted for radiomics nomogram from the training and validation cohorts (**Figure 5**). The AUC, classification accuracy, sensitivity, and specificity of radiomics nomogram were 0.937, 0.876, 0.907, 0.852 and 0.884, 0.831, 0.833, 0.829 in the training and validation cohorts, respectively. The calibration curves of the nomogram were shown in **Figure 6**. The calibration curves and the Hosmer–Lemeshow test showed good calibration in the training cohort (*P* = 0.697) and validation cohort (*P* = 0.244). The DCA result for the nomogram was shown in **Figure 7**. We found that using the multiparametric MRI model to predict LN metastases had a greater advantage when directing treatment decisions if the threshold probability was set between 0 and 0.8, compared with the treat-all-patients scheme and the treat-none scheme.

## Discussion

LN status is the key factor in determining whether to conduct adjuvant therapy or additional surgical resection (5, 7, 8). The accurate evaluation of LN metastasis using observable MRI features, such as size and morphology, remains challenging (34, 35). In this study, rad-score 3 was constructed that incorporated T2WI and ADC image features for preoperative prediction of LN metastasis in patients with rectal cancer and compared with the predictive performance of rad-score 1 based on T2WI features and rad-score 2 based on ADC features. The results indicated that rad-score 3 could yield the highest AUC score. We then developed and validated a radiomics nomogram incorporating rad-score 3 and some clinical information (age and LN size). The results showed that the model presented favorable predictive value for preoperative individualized prediction of LN metastasis in rectal cancer patients.

There have been some studies reporting the diagnostic value of radiomics in identifying the LN status of rectal cancer. Huang et al. (36) developed a radiomics model based on enhanced CT to predict LN status in colorectal cancer patients, and yielded an AUC score of 0.778 in the validation cohort. However, high-resolution MRI is regarded as the most common and effective method for the identification of clinical staging of rectal cancer (9). Several researches have shown that radiomics based on MRI had better diagnostic performance in discriminating LN status (21–23, 25, 26). An investigation by Yang et al. indicated that the histogram features from T2WI could be used to identify LN metastasis of primary rectal tumor and obtain the moderate-to-good diagnostic performance (AUC: 0.648 to 0.750) (21). A recent study demonstrated that radiomics model based on high-resolution MRI could be helpful in predicting LN status, which obtained an AUC of 0.8 in the validation cohort (23). In addition, DWI with ADC is a functional MRI sequence that can reflect the varying cellularity within a tumor (22). A study by Liu et al. showed that texture analysis on ADC maps could provide valuable information to predict LN status in patients with LARC (22). Recently, Zhou et al. established a radiomics model based on multiparametric MRI, including T1WI, T2WI, ADC, and CE-T1WI data, which yielded good diagnostic performance in predicting LN status for patients with LARC following neoadjuvant therapy (25). However, most previous radiomics analyses were generally performed with a single-slice image at the level of the largest section of the tumor. To improve the performance of radiomics models, three-dimensional VOI segmentation was conducted in our study. A prior study demonstrated that three-dimensional VOIs could contain more important information than two-dimensional regions of interest (37). Compared with those studies above, our research included more high-order features for radiomics analysis, such as square, square root, logarithm, exponential, gradient, and wavelet features. In addition, a recent study constructed a radiomics model based on VOIs of T2WI and DWI (b-value of 1,000 s/mm^2^), which achieved good diagnostic performance in the validation cohort (26). However, it was proved that texture analysis based on ADC maps achieved better discrimination performance to predict LN status than that based on DWI (b-value of 1,000 s/mm^2^) (38). Therefore, in our study, radiomic features were extracted from VOIs of T2WI and ADC images and used to establish a multiparametric model. Moreover, compared with these multiparametric MRI studies above, we also analyzed the discriminative ability of each imaging modality.

Rad-score 1, on the basis of T2WI, was mainly constructed by wavelet features (6/7); this demonstrated that wavelet features better reflected tumor biology and heterogeneity. All wavelet features were derived from the decompositions and the approximation by wavelet filter to the original image. Image transformation using a filter can eliminate noise or sharpen the image and does not change the semantics of the features (29). Therefore, these wavelet features represent the intensity distribution or gray level distribution of tumors in the corresponding wavelet filter image. For example, wavelet.LHL_firstorder_Maximum and wavelet.HHL_firstorder_Mean respectively describe the maximum and average gray level intensity of tumor region, wavelet.LHL_glcm_IDN is a measure of the local homogeneity of the tumor region and normalizes the difference between the neighboring intensity values by dividing over the total number of discrete intensity values, wavelet.LHH_glrlm_RE and wavelet.HHH_glrlm_RE represent the uncertainty/randomness in the distribution of run lengths and gray levels and a higher value indicates more heterogeneity in the texture patterns, and wavelet.HLL_gldm_LDLGLE measures the joint distribution of large dependence with lower gray-level values. The AUC of rad-score 1 for predicting LN metastasis was 0.749 in the validation cohort. One previous study also reported the effectiveness of wavelet features on T2WI in predicting LN status and obtained a similar result (39). Moreover, He et al. showed that wavelet features of T2WI had good performance in tumor grading for rectal cancer, which further demonstrated that wavelet features can reflect tumor biology and heterogeneity (40).

Rad-score 2, based on ADC images, was established by LOG, wavelet, logarithm, and exponential features. All higher-order statistics features derived from the image transformation using the corresponding filter could reflect underlying pathology information of the tumor. For example, log.sigma.5.0.mm.3D_glcm_IMC2 assesses the correlation between the probability distributions of two voxel spots in the log.sigma.5.0.mm filter image to quantify the complexity of the tumor texture, log.sigma.5.0.mm.3D_glrlm_LRLGLE measures the joint distribution of long-run lengths with lower gray-level values in the log.sigma.5.0.mm filter images, wavelet.LHL_glcm_Correlation quantifies the linear patterns in the wavelet.LHL filter image based on the distance parameter, wavelet.HLH_glszm_LALGLE represents the proportion in the wavelet.HLH filter image of the joint distribution of larger size zones with lower gray-level values, logarithm_firstorder_Median describes the average gray level intensity within the tumor region in the logarithm filter images, exponential_glszm_GLNU describes the variability of gray-level intensity values in the exponential filter image, with a lower value indicating more homogeneity in intensity values, and so on. A recent study showed a significant difference between texture features from ADC maps and LN metastasis status through statistical analyses (22). In our study, 11 higher-order statistics features from ADC maps exhibited highly discriminative performance, but six features were not significantly different between the LN-positive and -negative groups (using two-sample *t*-tests). We found that associating a single radiomic feature with complex tumor biological processes remained a challenge. Therefore, it was more common to combine the panels of selected features into a rad-score. Our results showed that the developed rad-score 2 could achieve good performance and yielded an AUC of 0.770 in the validation cohort. A recent study on breast cancer reported the potential values of higher-order statistics features in predicting sentinel LN metastasis (41).

Rad-score 3 was calculated by seven T2WI (six wavelet and one LOG features) and six ADC (two LOG, two wavelet, one logarithm, and one exponential features) features, and indicated that radiomic features on T2WI and ADC maps had good performance in predicting LN status. According to the AUCs, the rad-score 3 obtained the highest score among three rad-scores in predicting the LN status. Recently, several studies also reported that radiomics models based on multiparametric MRI data could improve the predictive performance for tumor characteristics (24, 31, 42, 43).

In multivariate logistic analysis, the rad-score 3, age, and LN size were identified as independent parameters of LN metastasis. We found that the LN positive group had a significantly younger age compared with the LN negative group which was consistent with that of the study conducted by Li et al. (44). This result showed that young patients with rectal cancer were more likely to have the risk of lymph node metastasis, which might be related to the high metabolism, dietetic irrationality, and lifestyle of young patients. In addition, as most young people lack the awareness of regular physical examination, the detection rate of rectal cancer in this population is low, which leads to the majority of patients in advanced stage and with a poor prognosis. However, our findings were in conflict with the study by Yang et al. (23), which concluded that no difference was observed in age between LN-positive and -negative rectal patients. This might be due to the different inclusion criteria of the study population between our studies. LN size represents the maximum short-axis diameter of regional LN. Several studies showed that LN size is an important clinical marker for the identification of LN status (25, 26). A radiomics nomogram incorporating rad-score 3, the age, and the LN size were developed. The results indicated that radiomics nomogram had good discrimination and calibration performance in both training and validation cohorts. Finally, the DCA showed that the model was clinically useful in the validation cohort.

A recent study showed that MRI radiomics based on multi-regions (peritumoral and intratumoral areas) could improve efficacy in the identification of LN metastasis in patients with rectal cancer (26). However, peritumoral tissue was not included in our analysis. That was due to the absence of uniform criteria for the peritumoral boundary. Another research demonstrated that the deep learning technology of faster region-based convolutional neural network could achieve excellent performance in discrimination, calibration, and clinical utility for preoperative identification of LN status (45). The performance of deep learning features was not investigated in our research, as this study focused on the feasibility of the radiomic features from the VOIs of T2WI and ADC features for LN status prediction. Therefore, to improve the performance of the prediction model, further work is expected to develop the model by combining radiomic and deep learning features based on multiregional MRI for preoperative prediction of LN status in patients with rectal cancer.

There were several limitations to this study. First, a bias of selection might exist because the study used a retrospective design. Second, the patient sample size was small and all cases were derived from a single institute. Multi-center studies with a larger sample set are required to further validate our model. Third, the segmentation of 3-D lesions was performed manually, which was time-consuming and complicated for the larger sample sizes. Thus, a fully automatic analysis method for rectal lesions with favorable reliability and reproducibility should be developed in further studies.

In conclusion, our study demonstrated that the radiomics nomogram, incorporating rad-score based on features from the T2WI and ADC images, and clinical factors, has potential for the preoperative identification of LN status. Although the results were satisfactory, the model should be validated by further studies with larger sample sizes from multiple centers to evaluate the performance.

## Data Availability Statement

The raw data supporting the conclusions of this article will be made available by the authors, without undue reservation.

## Ethics Statement

The studies involving human participants were reviewed and approved by Shengjing Hospital of China Medical University. The ethics committee waived the requirement of written informed consent for participation. Written informed consent was not obtained from the individual(s) for the publication of any potentially identifiable images or data included in this article.

## Author Contributions

CL conducted data analysis and manuscript writing. JY was responsible for the manuscript revision. All authors contributed to the article and approved the submitted version.

## Funding

This work was supported by the Research and development (R&D) foundation for major Science and Technology from Shenyang (No. 19-112-4-105), Big data foundation for health care from China Medical University (No. HMB201902105), Natural Fund Guidance Plan from Liaoning (No. 2019-ZD-0743), and 345 Talent Project from Shengjing Hospital of China Medical University.

## Conflict of Interest

The authors declare that the research was conducted in the absence of any commercial or financial relationships that could be construed as a potential conflict of interest.
