# Supplementary material for: Radiomics Based on T2-Weighted Imaging and Apparent Diffusion Coefficient Images for Preoperative Evaluation of Lymph Node Metastasis in Rectal Cancer Patients
Source: Front Oncol. 2021 May 10;11:671354. doi: 10.3389/fonc.2021.671354 (PMC8141802; doi:10.3389/fonc.2021.671354)
Supplement: Supplementary file 1 [file Table_1.docx]

**Table S1.** The features calculated with PyRadiomics using different radiomics analysis methods.

| **Methods** | | **Radiomic features** | **Number** | |
| --- | --- | --- | --- | --- |
| First order statistics | | Energy, total energy, entropy, minimum, 10th percentile, 90th percentile, maximum, mean, median, interquartile range, range, mean absolute deviation (MAD), robust mean absolute deviation (rMAD), root mean squared (RMS), skewness, kurtosis, variance, uniformity | | 18 |
| Texture | GLCM | Autocorrelation, joint average, cluster prominence, cluster shade, cluster tendency, contrast, correlation, difference average, difference entropy, difference variance, joint energy, joint entropy, informational measure of correlation1 (IMC1), informational measure of correlation 2 (IMC2), inverse difference moment (IDM), inverse difference moment normalized (IDMN), inverse difference (ID), inverse difference normalized (IDN), inverse variance(IV),  maximum probability(MP), sum entropy(SE), sum of squares(SS) | | 22 |
|  | GLRLM | Short run emphasis (SRE), long run emphasis (LRE), gray level non-uniformity (GLN), gray level non-uniformity normalized (GLNN), run length non-uniformity (RLN), run length non-uniformity normalized (RLNN), run percentage (RP), gray level variance (GLV), run variance (RV), run entropy (RE), low gray level run emphasis (LGLRE), high gray level run emphasis (HGLRE), short run low gray level emphasis (SRLGLE), short run high gray level emphasis (SRHGLE), long run low gray level emphasis (LRLGLE), long run high gray level emphasis (LRHGLE) | | 16 |
|  | GLSZM | Small area emphasis (SAE), large area emphasis (LAE), gray level non-uniformity (GLN), gray level non-uniformity normalized (GLNN), size-zone non-uniformity (SZN), size-zone non-uniformity normalized (SZNN), zone percentage (ZP), gray level variance (GLV), zone variance (ZV), zone entropy (ZE), low gray level zone emphasis (LGLZE), high gray level zone emphasis (HGLZE), small area low gray level emphasis (SALGLE), small area high gray level emphasis (SAHGLE), large area low gray level emphasis (LALGLE), large area high gray level emphasis (LAHGLE) | | 16 |
|  | GLDM | Small dependence emphasis (SDE), large dependence emphasis (LDE), gray level non-uniformity (GLN), dependence non-uniformity (DN), dependence non-uniformity normalized (DNN), gray level variance (GLV), dependence variance (DV), dependence entropy (DE), low gray level emphasis (LGLE), high gray level emphasis (HGLE), small dependence low gray level emphasis (SDLGLE), small dependence high gray level emphasis (SDHGLE), large dependence low gray level emphasis (LDLGLE), large dependence high gray level emphasis (LDHGLE) | | 14 |
| Square | | Filter images with square transform calculate first order intensity statistics and texture features | | 86 |
| Square root | | Filter images with square root transform calculate first order intensity statistics and texture features | | 86 |
| Logarithm | | Filter images with logarithm transform calculate first order intensity statistics and texture features | | 86 |
| Exponential | | Filter images with exponential transform calculate first order intensity statistics and texture features | | 86 |
| Gradient | | Filter images with gradient transform calculate first order intensity statistics and texture features | | 86 |
| Lapiacian of gaussian | | 3D Laplacian of Gaussian (LoG) filtering by changing sigma values to 5.0, 4.0, and 3.0 mm yields 3 derived images, calculating first order intensity statistics and texture features in the per derived image respectively. | | 172 |
| Wavelet | | Wavelet filtering yields 8 decompositions per level (all possible combinations of applying either a High or a Low pass filter in each of the three dimensions [x, y, z]), calculating first order intensity statistics and texture features in the per decomposition image respectively. | | 688 |

NOTE. GLCM, Gray Level Cooccurence Matrix; GLRLM, Gray Level Run Length Matrix; GLSZM, Gray Level Size Zone Matrix; GLDM, Gray Level Dependence Matrix

**Table S2.** Radiomics features associated with pathology outcome identified by two sample t-test and LASSO

| Radiomics Feature | | Normalized values  (mean ± standard deviation) | | | | *P* | | Number | Coefficient in  SVM model | |
| --- | --- | --- | --- | --- | --- | --- | --- | --- | --- | --- |
|  |  | LN (+) | | LN (-) | |  |  |  |  |  |
| T2WI | | |  | |  | |  |  | Rad-score1 | Rad-score3 |
| log.sigma.5.0.mm.3D_glcm_DE | -0.3635 ± 0.8963 | | | 0.2675 ± 0.9514 | | | *0.001* | T1 | -0.4056 | -0.3491 |
| wavelet.LHL_firstorder_Maximum | -0.3233± 0.8602 | | | 0.3170 ± 1.0683 | | | *0.002* | T2 | -0.1705 | -0.1203 |
| wavelet.LHL_glcm_IDN | -0.4047 ± 1.2387 | | | 0.2154± 0.7689 | | | *0.003* | T3 | -0.3491 | -0.4237 |
| wavelet.LHH_glrlm_RE | -0.3329 ± 0.9377 | | | 0.3206 ± 0.8873 | | | *0.001* | T4 | -0.0113 | -0.3215 |
| wavelet.HLL_gldm_LDLGLE | 0.4790 ± 1.3027 | | | -0.1607 ± 0.7816 | | | *0.003* | T5 | 0.2231 | 0.2928 |
| wavelet.HHL_firstorder_Mean | 0.3464 ± 1.0754 | | | -0.2689 ± 0.9655 | | | *0.004* | T6 | 0.5804 | 0.5249 |
| wavelet.HHH_glrlm_RE | -0.4041 ± 1.1135 | | | 0.3286 ± 0.7945 | | | *<0.001* | T7 | -0.4599 | -0.1402 |
| ADC |  | | |  | | |  |  | Rad-score2 |  |
| log.sigma.5.0.mm.3D_glcm_IMC1 | -0.3069 ± 1.1005 | | | 0.2743 ± 0.7311 | | | *0.002* | A1 | -0.3790 | 0 |
| log.sigma.5.0.mm.3D_glcm_IMC2 | 0.3532 ± 0.8182 | | | -0.3035 ± 1.0126 | | | *0.001* | A2 | 0.1614 | 0.1051 |
| log.sigma.5.0.mm.3D_glrlm_LRLGLE | -0.2057 ± 0.8336 | | | 0.1620 ± 0.8856 | | | *0.040* | A3 | -0.9420 | -0.8348 |
| wavelet.LHL_glcm_Correlation | -0.2565 ± 1.0965 | | | 0.0951 ± 0.8888 | | | *0.084* | A4 | -0.5929 | -0.3617 |
| wavelet.LHH_glcm_IDMN | -0.2164 ± 0.9717 | | | 0.1111 ± 0.8881 | | | *0.087* | A5 | -0.1088 | 0 |
| wavelet.HLH_glszm_LALGLE | -0.2259 ± 0.4346 | | | 0.2039 ± 1.3100 | | | *0.042* | A6 | -0.1166 | -0.3024 |
| logarithm_firstorder_Median | 0.2986 ± 0.9792 | | | -0.2656 ± 0.9993 | | | *0.006* | A7 | 0.4203 | -0.0844 |
| logarithm_glszm_SZNU | -0.2230 ± 0.7052 | | | 0.0677 ± 0.9327 | | | *0.094* | A8 | -0.3066 | 0 |
| exponential_glcm_IV | 0.1726 ± 0.9247 | | | -0.1744 ± 1.0786 | | | *0.097* | A9 | 0.4202 | 0 |
| exponential_glszm_GLNU | -0.2052 ± 0.9400 | | | 0.1303 ± 0.8947 | | | *0.076* | A10 | -0.5737 | -0.2718 |
| exponential_glszm_LALGLE | -0.2856 ± 0.5512 | | | 0.1833 ± 1.2204 | | | *0.022* | A11 | 0.2310 | 0 |

Abbreviations: ADC, apparent diffusion coefficient; T2WI, T2-weighted images; glcm, gray-level cooccurrence matrix; glrlm, gray level run length matrix; glszm, gray level size zone matrix.

**Rad-score1 based on T2WI features calculation formula:**

Rad-score1 = - 0.2379 - 0.4056 × T1- 0.1705 × T2 - 0.3491 × T3 - 0.0113 × T4 + 0.2231× T5 + 0.5804 × T6 - 0.4599 × T7

**Rad_score2 derived from ADC maps calculation formula:**

Rad-score2 = - 0.2428 - 0.3790 × A1 +0.1614 × A2 - 0.9420 × A3 - 0.5929 × A4 - 0.1088 × A5 - 0.1166 × A6 + 0.4203 × A7 - 0.3066 × A8

+ 0.4202 × A9 - 0.5737 × A10 + 0.2310× A11

**Rad-score3 based on T2WI and ADC images features calculation formula:**

Rad-score3 = -0.2342 - 0.3491 × T1 - 0.1203 × T2 - 0.4237 × T3 - 0.3215 × T4 +0.2928 × T5 + 0.5249 × T6 - 0.1402 × T7 + 0.1051 × A2

- 0.8348 × A3 - 0.3617 × A4 - 0.3024 × A6 - 0.0844 × A7 - 0.2718 × A10
